# On the Use of Tartar Emetic, Etc. in Epidemic Dysentery

**Published:** 1850-01

**Authors:** 


*On the use of Tartar Emetic, etc., in Epidemic Dysentery.* By Samuel Jackson, of Philadelphia, late of Northumberland.

Tartar Emetic has lately been considered by very high authorities as dangerous to children, by suddenly prostrating the system; and the frequent repetition of this medicine is denounced by them as dangerous in any age, by superinducing a fatal follicular enteritis. This is a comparatively novel discovery, and a transient bubbling up of the sinking, putrefying doctrine of Broussais.

"——— alto

Demersus, summa rursum nunc bullit in unda; it is not to be supposed that such a man can sink like the poet's blockhead, without a bubble on the surface. But what this medicine does now, it must have done heretofore; let us see then what authors have said and left for our instruction. But first let me relate a portion of my own experience, for it is the result of theirs.

I passed through ten tremendous epidemics of remittent fever in Northumberland, besides prescribing for many sporadic cases in other years; in almost every case I gave small and frequent doses of tartar emetic during nearly the whole course of each case. My practice was to carry papers of different sizes in my pocket, and to dissolve them in a proper number of table-spoonfuls of water, according to the wants of the family, giving a spoonful every hour or two, containing, for the adult, from one-sixteenth to the one-eighth of a grain. Beginning in this way, very little nausea was produced, and the medicine could be often increased to one-quarter of a grain. This was an ever ready and cheap medicine, soon prepared and easily given, costing the family that inestimable something vulgarly called *nothing*—when in truth it is a mine of gold and often worshiped in the doctor's bill—to use an Irishism—as the fourth person in the Trinity.

"Nam *nihil* est gemmis, *nihil* est pretiosius auro,
Diique *nihil* metuunt. Quid longo carmine plura
Commemorem? virtute *nihil* præstantius ipsa
Splendidius *nihil* est, *nihil* est Jove denique majus."

I kave attended many thousand cases of remittent fever, I am afraid to say how many, in all ages, from one month old to eighty years, and I do not believe that many of them escaped my solution of tartar emetic. My success was such that I have continued in the practice, always greatly surprised when I hear it denounced, and ready to say—you know not how much good you withhold from your patients.

It is a fashionable notion in great cities, and particularly among the physicians, that a country doctor can see but little practice, this is probably the greatest mistake the poor cockneys labor under, for in all other respects they are truly most clever men, whom I would delight to honor were it in my power. In this particular they greatly need information. Let them know, then, that a country physician in the centre of a village of a thousand or more inhabitants, all of whom are his patients, when sickness invades, has such facilities of visiting at any hour, day or night or morning, that he can see more patients before breakfast *in an epidemic fever* than a city physician can see dring the twenty-four hours. Then he takes his circuit in the country, and finally visits his patients in town during the night. It is only to step from house to house—"*intervallum facile commodum*, as Horace says, but in no satirical sense.

Now add to my remittent fevers all the cases of other inflammatory diseases, which I have attended for thirty-eight years, in not one of which had I reason to suppose that the poor gastro-intestinal-mucous membrane suffered in the least from the medicine. Nor was I very inattentive to the subject, for the panic of the Broussaians pervaded our rustic minds, of course, upon hearing from Philadelphia that the physicians were so alarmed for the safety of this *delicate* organ, that they would not suffer their dogs to eat bones, nor their horses to eat hay—trembling for an organ that digests masses of iron or copper, and resists the impressions of ether and brandy.

In all inflammatory diseases in which I gave the tartar emetic, I was encouraged by its uniform effects to give it again. Its simultaneous operation on the bowels and skin were very beneficial, and had no equal in any other medicine. Add to this the testimony of Dr. Lind, that tartar emetic exerts a febrifuge virtue independent of any evacuation; and this I know to be true to nature, for hundreds of times have I seen fever dying away under its use, without sweat or purging, as calmly as the day dies away with the setting sun. One year I tried ipecacuanha in small doses, hoping it would operate by diaphoresis and gentle purging, but my cures were slow, and my patients therefore accumulated so greatly that I was obliged to recur to tartar emetic.

My general plan was to bleed freely, give from ten to fifteen grs. calomel at bedtime, and a dose of some active purgative, generally oil, very early in the morning, so as to have the severity of the purging done during the remission. The exacerbation in the afternoon was thus not only mitigated, but it was met by the tartar emetic, and often most happily turned into a diaphoresis. In this I desired neither vomiting nor nausea, and if either occurred, a few drops of laudanum would turn the operation of the tartar to the surface. All this is most beautifully displayed by Lind on Hot Climates, art. Fever—sub principio.

I cannot say that I have never given this medicine too freely, and never thus superinduced some present unnecessary and improper distress; but this I considered as my own fault and not that of the medicine—a fault that I hoped others would anoid and myself eschew in the future. But did no anti-tartar doctor ever give too much of any other medicine? It is vain to answer in the affirmative, for all active medicines are sometimes abused by the best of physicians, even by the *Physiologists* themselves, owing to some incrutable idosyncracy, or some *incuria*, which Horace says, human nature can hardly avoid.

Some misfortunes of this kind must have happened to a great practitioner whom I have heard declaim with unusual vehemence against this medicine in dysentery, wherein he said it would produce follicular inflammation and death. This gentleman, as well as some who have written in this strain, must have been very unfortunate, for they are considered as too philosophical to infer so much from a *few* observations or slender premises. If each of them has been in the practice of giving tart. emet. and has thereby brought on fatal follicular enteritis, and has dissected a sufficient number of his patients, from whom to deduce philosophically an important fact, he ought to tell us plainly what he has seen and done. This is now the only atonement he can make; and let him be assured it will be received as full satisfaction by the medical profession, which has always been highly in favor of reporting unfortunate cases. But what say authors of the first authority with respect to the use of this medicine in dysentery?

Cullen says "that if gentle laxatives do not answer, some more powerful medicine must be employed; and I have found nothing more proper or convenient than tarter emetic given in small doses and at such intervals as may determine their operation chiefly by stool."—*First Lines*, § 1079.

Cleghorn says, that of the vitrum ant. cerat. "I directed ten or fifteen grs. in powder to be divided into three doses, and to be taken in the forenoon, at the interval of two hours, or an hour and a half. The most common effect of both was, to procure a thorough evacuation upward and downward during the day, and they often threw the patient into a sweat the ensuing night." I must acknowledge that now and then, in desperate bloody fluxes, I have known the antimonial medicine to be successful after every thing else had been tried to no purpose." For the first three or four days, he repeats these evacuations every other day, and afterwards at greater intervals, giving a small dose of opium to procure rest and promote perspiration.—*Rush's Cleghorn, p.* 146–7.

Donald Munro, second edition, vol. i. 339 to 342 inclusive, contains much to our present purpose. After premising bleeding and vomits of ipecac, he says, "when the patient was strong, and he wanted to make a free evacuation, we added one, two, or three grs. tart. emet. which commonly operated by stool; and since my return, I have frequently given with advantage a gr. of tart. emet. dissolved in two ounces of water, every quarter of an hour till it vomited freely, or both vomited and purged the patient." He says also, that Mr. Russel of the hospital of Martinico, pursued this very method in his own practice. He adds, moreover, that Dr. George Munro and Mr. Russel, both of the military hospitals during the war in North America, gave tart. emet. and manna in small and frequent doses till they vomited and purged, " and this commonly produced good effects."

Dr. John Clark—Dis. of Long, Voy, vol. ii. 325,—says he gave vomits of tart. emet. and ipecac, in dysentery; but he preferred the tart. emet. alone when the patient was feverish; and it not only proved one of the most powerful emetics, but, by likewise acting as a purge, it relieved the tenesmus." He says further, page 339, that he had often given with good effects a quarter grain tart. emet. with ipecacuanha. This last medicine he considered as of no value when unassisted by the tart. emet.

Dr. Darwin is a writer to whom I shall always refer with peculiar pleasure, for I derived more instruction from him in my early practice than from any other author. He recommends *antimonials*, by which he means, as his readers well know, the tart, emet. in small and frequent doses.

Pringle commends vit. ant. cerat. in dysentery, but desisted from the use thereof as he says, through the prejudices of others: he then used ipecac, and added one or two grs. of tart. emet. Dr. Huck, another highly gifted physician of the same army, see Rush's Pringle 236, pursued the same practice; and M. De Senac, then Physician General to the French army, informed Pringle " that after evacuating by bleeding, and by a vomit of emetic tartar, his practice consisted chiefly in giving one grain of that antimonial preparation dissolved in a pint of common whey or chicken water, in divided draughts every day for all food, drinks and medicine, till the patient recovered. His intention, he says, was to keep a free passage from the stomach to the rectum by the mildest laxative, which he found was that minute quantity of the emetic.,'

Sir George Baker, I quote from Moseley, gave a vomit in the beginning and preferred tart. emet. to ipecac. He knows no virtue in ipecac, except that of its emetic property, wherein it is not equel to tart. emet. He says if Friend found ipecac. useful on account of its diaphoretic property, he can assure us that in this it is not equal to the tart. emet. This, moreover, he says, operates downwards, and he supposes that the vit. ant. owes its anti-dysenteric reputation to its emetic and purgative operations.

Dr. Chapman in his Therap. says, that he prefers ipecac. in dysentery to tart. emet..; but he says also that great deference is due to the authority of such men as Pringle and Baker. Dr. C. gives directions for its administration in dysentery, and hence he must approve of its use. He must surely consider it a very mild and manageble medicine, since he gave it in the form of antimonial wine, as he tells us. loc. cit. to a child as soon as born for the purpose of vomiting.

Dr. Mosely, see 4th edition, 1803, from page 247 251, founds his doctrines on his experience in the army of Jamaica. After free and sometimes repeated bleedings, he gave a vomit of ipecac. and then an "antimonial that would operate both on the bowels and skin. Then "the *primæ viæ* being cleansed and the revulsion begun, it must be supported by sudorifics, that the disease may be thrown off by sweat; this will be effected by uniting an opiate with a diaphoretic, and administering it as occasion requires. Laudanum and antimonial wine combined, is a medicine that causes little or no irritation and is a pleasant and certain diaphoretic."

He then commends James' powder for the same purpose, and speaks strongly of the necessity of keeping the patient well covered, so that a continual diaphoresis may be maintained. At p. 240, 241, he considers that tart. emet. in many respects is a dangerous medicine *in hot climates.*" "As it acts immediately on the stomach, it is frequently impossible to produce any other effect by it in whatever dose administered." He seems not to have known that his favorite antimonial wine is this very medicine thus hastily condemned; nor does he seem to have known that James' powder is a far more unmanageable powder than tart. emet.

Moseley's practice, then, in the West Indies, and afterwards in London, was to attack the disease most vehemently by bleeding, sometimes repeated, followed by an emeto-cathartic of James' powder, or vit. ant.—see p. 270, 277—finishing the disease by a long continued sweat and catharsis at the same time, maintained by antimonial wine *alias* tart. emet., turned in part to the skin by laudanum, warm drinks, blankets, and seclusion from the air. Is any man is mad enough to call this madness, he must admit "there is method in it." What says the venerable Dr. Nathan Smith?

"Since I first read his (Dr. Moseley's) book, the dysentery has been epidemic several times, and I have had frequent opportunities of contrasting his mode of treatment to taat of others, and am decidedly of opinion that it is the best I have been acquainted with." Again he says, "several judicious practitioners have declared in its favor." *Note to his edition of Wilson on Feb. Dis. p.* 389.

Tissot, says "the great remedy is an emetic." His No. 34, (six grains tart. emet.) "when there is certainly no objection to its use, if taken early, often carries off the whole disease, ane never fails to shorten its course." He says the disease made its greatest ravages in towns wherein the people rejected emetics. For malignant dysentery he gave ipecac as a principal remedy. He first puked with it, then purged with rhubarb, and lastly gave ipecac in small frequent doses, no doubt with a view to diaphoresis. *Avis au peuple,* vol. ii.

Now let us see what that orthodox *physiologist,* Dr. John D. Godman, used to do. In the Ph. Jour. of the Med. and Ph. Sci., vol. xii. 197, we read over his own name that he gave the tart. emet. mixed with flaxseed tea; and this in a "dysentery that rapidly prostrated their strength by the great number of bloody mucous discharges and continual febrile irritation." Dr. Rush used to say "that a bad theorist was often a good practitioner." This is no doubt true, for experience will triumph over theory at the bedside.

Dr. Thos. Worthington, Hartford county, Maryland, in a letter to the editors of Med. Record, vol. xiv. 199. writes that Dr. Thos. Bond, of Baltimore, recommended to him the vit. ant. He gave it in doses of one grain every four hours, determining its action to the skin and bowels by diluents and an occasional dose of calomel. "This preceded by an emetic and assisted by mucilaginous drinks—sometimes by bleeding, enemata, fomentation and blisters—constituted the outline of treatment for several years that was attended with unfailing success."*

Professor Wood, of the University of Pennsylvania, says in his *Practice of Medicine,* "when the skin is hot and dry, small doses of the tart. emet. or the neutral mixture.may be given separate or combined, at intervals of an hour or two." This invincible authority ought to have been mentioned sooner, and where it would have been more certain of a reader, but the previous sheets are now gone to the press.

Dr. O'Brian, (Observations on the Dysentery of Ireland) thinks that emetics have undeservedly fallen into disuse, hence he gave antimonial powder, with his calomel and opium. Speaking of sudorifics and purgatives combined with opium, he says: the "mercurial salts, Dover's powders, and James's powders are the principle medicines of this class applicable to the cure of dysentery. Four or five grains calomel with as much James' powders, and a grain of opium may be given every third or fourth hour, or even more frequently." p. 45.

Le Dict. des Scien. Med. Art. Dysent. CXLIX says, "that the evacuation of the alimentary canal is not the only advantage procured by vomits, they

*The sequel of this letter I shall notice hereafter.

commonly determine to an abundant diaphoresis. The tart. emet. effects this even when it acts as a gentle purge; and we often see dysentery, even whenfully formed, to be carried off immediately by vomits. They ought to be seldom repeated, except the tart. emet. which is often given in very small doses diluted with drinks; then it proves to be only a mild diaphoretic and gentle purge." In cxcv (on infl. dysent.) the author says: "Emetics and purges must prove hurtful by irritating parts already excessively inflamed; but tart. emet. mixed with drink—a grain in two or three pounds of liquid—not acting as a vomit, produces a mild diaphoresis always favorable. If some gentle stools are obtained from this medicine, they are without irritation. But much experience is necessary in the use of this remedy."

In ccxiii, *on gastric and bilious dysentery*, he says, "we must begin the treatment by vomits, for bleeding is injurious. We shall give the preference to tart. emet. taken in small frequent doses; and it is generally useful to repeat them." Then come purgatives, and he recommends among others the calomel, as it never irritates the intestines.

Enough has now been quoted to show that antimony is a remedy in dysentery not to be rashly condemned. The above authorities are some of the highest in the world, and many others might no doubt have beee found; but time is wanting, and moreover, they are not needed. Have we not now a right to require that physicians who, from theoretical views, declaim against this medicine as producing *follicular enteritis*, shall make a fair trial thereof and report their dissections? I was taught the use of tart. emet. in *fevers* by the great Rush and the illustrious authors whom he was accustomed to quote; I have been using it freely for thirty-eight years, and am now ready to say to its opposers—you know not how much good you withhold from your patients.

I will not say that it never inflames the mucous memprane, for I think it very possible that like Fowler's solution, it may do this when perserved in by theoretical rashness and folly: but I have been so fortunate as never to have seen this evil either in my own practice or in that of others. As mentioned above, I have given it in remittent fever to thousands of all ages, from one month old to 80 years, and without any striking case to be regretted, for I do not remember one. So safe was the practice that the Northumbrians used to procure the tartar from the apothecary and cure their fevers as they had often seen their physician do in similar cases. I have not used it in dysentery as often as my late experience teaches me that I ought to have done, and this was the very reason of my bringing it before the Society at their late meeting; for hearing nothing of the practice now advocated, I supposed others like myself, had too much neglected it.

Should the authorities quoted above induce any one to try this medicine in dysentery, let him do it fairly and *with all all its coadjuvants*. We are not recommending a single and infallible remedy in the manner of the quacks. Bleeding and leeching must be carried as far as the patient's strength will admit; and it will bring down the pulse to the diaphoretic grade. The patient is now in a perspiration, the violence of the disease is broken, and he is fairly on the road to health. This state of things must be maintained by small frequent doses of tart. emet. the patient covered witn a blanket and constantly under the mild diaphoretic and anodyne influence of opium. If sweating is not steadily maintained, much of its virtue will be lost, though still some good may be effected through its operation on the bowels, and through that mysterious action by which Lind, Fordyce, Chapman, and others say and say truly—it annihilates fever without either purging, puking, or sweating. But the dose must be increased as the stomach is found to bear it without nausea, for just in proportion to the quantity taken will be its evident febrifuge effects. I cannot certainly say with Fordyce and Chapman,* that nausea and vomiting counteract its just operation on the fever, but this distress of the stomach is not tolerated by the patient, and the medicine is thrown out at the window in disgust not easily forgotten, and sometimes hardly forgiven. Beginning with one sixteenth of a gr. every hour, you may gradually increase it to one-sixth or even one-fourth, and this is strong enough to fulfil every possible indication.

But it must not be forgotten that other means are required; that venesection must sometimes be repeated, and leeches also, if the pulse, the strength, and the *vivida vis* will admit thereof. The physician must fix his mind on the inflammation, remembering that this is the sole cause of death. Opium is a feverish stimulant and a great evil, but fortunately the tartar emet. may determine this to the skin, and thus change its evil into good. If this is beautiful in theory and also on paper, it is infinitely more so in practice, and for its truth the reader is referred to the authors quoted above; though not one of them except Mosely appears to me to have derived from the medicine its ultimate good. There is little said by the others on maintaining a mild perspiration on which the resolution mainly depends. If the patient is permitted to rise to the chair or lie half naked in his impatience of heat, the perspiration is checked and the disease renewed. But bleeding is the present subject, and to this we recur.

Some writers are opposed to this, and some recommend it with great timidity. In idiopathic fevers you may bleed away the inflammation and yet the fever may not be resolved—it will remain and kill the patient, nor can necrotomy reveal the cause; but dysentery is as much a local disease as peritonitis, pleurisy, or encephalitis from a stroke of the sun—resolve the inflammation and the patient is cured. As to the debility it superinduces, the question is—whether the patient is more dangerously broken down by losing blood from the arm than by the long continuance of a bleeding tenesmus and the tedious process of nature's operations. But it cannot be made a question whether heat or climate or the air of an hospital forbid this remedy or render it less necessary, as we may learn from Moseley and many of the English physicians in India whose authority can not be resisted.

It is perhaps in young children that bleeding and tart. emet. are most necessary, for to them the disease is more fatal than to adults, through an excess of inflammation; and to them these remedies are more easily applied than blistering, leeching, and other apparatus.

Bat while we advocate the above strenuous debilitation, we are not unadvised that there are many cases of common dysentery that are easily cured by the same means very gently used; it is always safest, however, to con-

See Fordyce, 3d. Diss. part 2d., and Chapman's Therap.

sider that a mild case may become severe until it be brought fairly under the influence of medical treanment.

Nor are we ignorant of the fact, that in every climatc there are sometimes sporadic cases and often whole epidemics, which bear very little if any medical debilitation. Of this Dr. Worthington, whom we have already quoted—Med. Record. xiv. 199—give a remarkable example in our own country. After curing common dysentery by vit. ant. for many years, and to his entire satisfaction, he had, in 1825, the mortification to find this medicine "to fail entirely." Tart. emet. and calomel were uniformly injurious, and the patients were cured by camphor and opium, the bowels being frequently opened by Epsom salts.

It is in such cases and such epedemics that spirits of turpentine would no doubt prove of pre-eminent service; for in common inflammatory dysentery, when the vigor of the inflammation is broken and the system will not bear much further depletion, but particularly when the tongue has cleaned off and now become dry and red, the mucous membrane probably a little tending to ulceration—then this medicine, both internally and externally, is almost uniformly beneficial, and sometimes works like a charm. Here the inflammation has been brought to that *passive state*, to use an obsolete word, which probably exists in what has been called typhoid dysentery.

There is something almost marvelous in the operation of this singular medicine. In many inflammations of debility, as in that of the mcuous membrane in typhus and typhoid fevers and dysentery, in the peritonitis of low puerperal fever, in burns and scalds, it would seem to have some specific power. I am not writing for the novice, and therefore, without descending to particulars, I may here dismiss the subject. I brought it before the Scoiety with the hope of hearing some favorable reports from the eminent practitioners present.

Another subject which I brought before the Society for discussion was—whether emollient and anodyne injections or the same with sac. sat. had been found useful. When I was a student of medicine in Philadelphia, I was taught that injections of every kind were to be avoided in this disease, as generally adding to the present irritation, and hence I do not recollect ever using them in my country practice. Finding them used on my return to the city, I tried them with the event of confirming my early prejudice. Laxative injections are seldom needed, for there is nothing in the lower bowels to be removed; and the anodyne, however small, are generally expelled with pain and tenesmus. I recollect they are applauded by some writers and rejected by others, but I have not leisure to make researches. Broussais—Hay's translation, vol. ii. 248—says, "as to enemata of mucilage, of oil, of bran, tripe water, &c. I look upon them as foreign bodies which, by forcibly dilating and irritating the suffering membrane, are most frequently injurious." Laxative enemata, however, he permits, when it is known that the lower bowels are filled with matter more irritating than the enema itself, a state of things that never happens. Dr. James Johnson, a great authority, discards them altogether, and so do others to whom I cannot at present refer with certainty.

The late practice of using strong injections of nitrat. arg. is very plausible in theory, as they may instantaneously cauterize the sensible parts: and though they be immediately expelled, their work is done, Of these I have no experience except in chronic cases.

Another subject I introduced for discussion was, whether butter-milk had been used by any of the members, as an article of diet in dysentery. This was brought into use by Dr. J. Young, of Chester, Del. county, and mentioned by prof. Wood in his Pract. of Med. Dr. Young—Amer. Jour. Med. Scien. new series, vol- vi. 259, published in 1842—said that he had then used it largely for twelve years, and with surprising advantage. He permits his patients to drink at the rate of a gallon in twenty-four hours, if such be their desire. In the summer of 1848, Dr. Tucker and myself, in a very dangerous case of great importance, made our first trial, and it proved of decided benefit. Since that time I have often used it, always with entire satisfaction, and frequently with decided advantage. When Dr. Wellford, of Virginia, was lately in this city he said, that he should always thank me for writing to him on this subject; for he found the buttermilk of great benefit in dysentery, and that it had, as he believed, enabled him to save the life of an inestimable patient.

When practising in the country, where this article was always at hand, I well remember that my patients preferred it, as made into a porridge with flour, to any other food; and they would continue to eat it with pleasure for a long time, calling every other article of low diet by the opprobrious name of slops. Fresh unsalted butter, as every one knows, has been long used; but its benefit was supposed to result from its purgative property. It is very probable that the utility of buttermilk is derived in part from the infinitesimal particles of butter that remain diffused through it; hence I have supposed that the whole mass of churned cream might be taken from the churn before the butter has yet been agglomerated, and thus used as diet and medecine. A diminutive churn could be made, by which two or three quarts of milk might be extemporaneously converted by any family into this wholesale and medicinal beverage.

The above suggestions and inquiries have for the most part resulted from my own practice during the last few years. They were brought before the Society for the purpose of exciting discussion, according to the order recently established thereby. And now, should any good natured dodger, rather disposed to yield than contend, be induced to read thus far, he will probably say—"this antimonial practice may answer with some robust patients, but I don't approve of carrying it too far—I would not give too much. ' Let us assure him, that we are equally averse to this *too much*, which is the truant and unmeaning phrase of many. The practice with small doses of tart. emet. is purely tentative, and will not be overdone by the young practitioner. The French author quoted above says, that "much experience is necessary in the use of this remedy." Perhaps there is not so much necessary in giving tart. emet. as in drawing blood in this disease. When the vital fluid is poured out, it cannot be returned; but in giving the tart. emet. in such small doses, repentance may come in time to prevent all mischief.—*Medical Examiner*.

